# Association between Dietary Fiber Intake and Hyperuricemia among Chinese Adults: Analysis of the China Adult Chronic Disease and Nutrition Surveillance (2015)

**DOI:** 10.3390/nu14071433

**Published:** 2022-03-30

**Authors:** Qianrang Zhu, Lianlong Yu, Yuqian Li, Qingqing Man, Shanshan Jia, Yonglin Zhou, Hui Zuo, Jian Zhang

**Affiliations:** 1 National Institute for Nutrition and Health, Chinese Center for Disease Control and Prevention, Beijing 100050, China; zhuqianrang@hotmail.com (Q.Z.); lianlong00a@163.com (L.Y.); cnu_lyq@126.com (Y.L.); manqq@ninh.chinacdc.cn (Q.M.); jiass@ninh.chinacdc.cn (S.J.); 2 Jiangsu Provincial Center for Disease Control and Prevention, Nanjing 210009, China; jsepipublic@sohu.com; 3 School of Public Health, Medical College of Soochow University, Suzhou 215123, China; zuohui@suda.edu.cn

**Keywords:** dietary fiber, cereal fiber, serum uric acid, hyperuricemia, China Adult Chronic Disease and Nutrition Surveillance

## Abstract

This study aimed to assess the association of dietary fiber intake with serum uric acid (SUA) levels and risk of hyperuricemia (HUA) among Chinese adults using the latest nationally representative data. A total of 66,427 Chinese adults aged 18 years and over from the China Adult Chronic Disease and Nutrition Surveillance in 2015 were included in this study. Dietary intakes were measured with a 3-day 24 h dietary recall and the household condiment weighing method. Mixed-effect linear and logistic regression models were used to evaluate the associations of dietary fiber intake with SUA levels and risk of HUA, respectively. Compared to the lowest intake group, the coefficient and 95% confidence in the highest intake group of total fiber were −0.06 (−0.08, −0.04) (*p*-trend < 0.001), −0.18 (−0.2, −0.16) (*p*-trend < 0.001) for cereal fiber, 0.03 (0.01, 0.04) (*p*-trend = 0.051) for legume fiber, 0 (−0.01, 0.02) (*p*-trend = 0.869) for vegetable fiber and 0.01 (−0.001, 0.04) (*p*-trend = 0.296) for fruit fiber. The odds ratio (OR) and 95% confidence interval (CI) of HUA for the highest vs. lowest intake group of total fiber were 0.88 (0.84, 0.91) (*p*-trend = 0.001), 0.67 (0.63, 0.71) (*p*-trend < 0.001) for cereal fiber, 1.05 (1, 1.09) (*p*-trend = 0.248) for legume fiber, 1.01 (0.97, 1.05) (*p*-trend = 0.982) for vegetable fiber and 1.06 (1, 1.12) (*p*-trend = 0.264) for fruit fiber. Our findings suggest that consumption of total fiber and cereal fiber were significantly inversely associated with SUA levels and HUA risk among the Chinese adult population. Developing and implementing effective public education programs are urgently needed to increase the intake of dietary fiber, especially cereal fiber among Chinese adults.

## 1. Introduction

Hyperuricemia (HUA) is a chronic metabolic disease, characterized by abnormally elevated serum uric acid (SUA) levels, caused by increased production and/or decreased excretion in uric acid [1]. HUA has always been considered a main risk factor for gout due to accumulation of circulating urate and crystallization, and is present in almost all gout patients [2,3]. HUA might also be involved in the development of some chronic diseases, such as obesity, diabetes, hypertension, cardiovascular disease and chronic kidney disease [4,5,6,7,8]. In recent years, HUA has been increasing in prevalence in many countries, and has become an urgent public health problem worldwide [9]. It is estimated that approximately one in every five American adults suffered from HUA in the year 2015–2016 [10]. In China, the overall prevalence of hyperuricemia for adults reached 13.3%, more than 180 million patients, in 2014 [11]. In addition, several European countries, including Italy, Switzerland, Russia and Ireland, have hyperuricemia prevalence rates of 9% or above, with Ireland’s prevalence reaching 24.5% in 2014 [12,13]. Several lifestyle modifications, including weight control, regular physical activity, restricting alcohol intake and sugar-sweetened drinks as well as meat and seafood are widely recommended for the management of HUA and gout [14].

Dietary fiber refers to a heterogeneous group of non-digestible plant polysaccharides found in high amounts in fruits, legumes, vegetables and cereals. Dietary fiber is considered an essential healthy component in the diet and has been shown to reduce the risk of some metabolic diseases such as dyslipidemia, hypertension, obesity and diabetes [15]. SUA levels are determined by the balance between hepatic production of urate and intestinal or renal urate excretion pathways [16], and thus may be mechanistically associated with dietary fiber intake, which has the potential to improve renal function as well as increase gut transit rate and fecal bulk [17,18]. Several animal studies have shown that dietary fiber significantly inhibits the increase in SUA concentrations induced by dietary ribonucleic acid, adenosine-50-monophosphate, adenosine, or adenine and suppresses experimental HUA in rats [19]. Cross-sectional studies among the U.S. population and Chinese population in Taiwan have reported that dietary fiber intake to be inversely associated with SUA concentrations [20,21,22] and HUA risk in adults [22,23]. However, a cross-sectional analysis found there to be a negative correlation between dietary fiber intake and SUA concentration only in Australian participants, but not in Norwegian participants, despite all being white Caucasians [24]. 

To date, the epidemiological evidence on this issue is still limited and inconsistent. Furthermore, the effects of different sources of dietary fibers on SUA levels remain unclear. Therefore, this study aimed to examine the associations of dietary intakes of total, cereal, legumes, vegetable and fruit fiber with SUA levels and risk of HUA in Chinese adults by using the latest nationally representative data.

## 2. Methods

### 2.1. Study Design and Participants

Data were obtained from the China Adult Chronic Disease and Nutrition Surveillance (CACDNS) 2015. The CNCDNS is a national representative cross-sectional study conducted by the Chinese Center for Disease Control and Prevention to assess food and nutrient intakes, current chronic disease status and health behaviors in the Chinese population. This survey was conducted at 302 monitoring sites across 31 provinces (except Hong Kong, Macau and Taiwan) in China. As has been previously described in detail, both multistage stratified cluster random sampling and probability proportionate to population size sampling methods were employed in this survey [25]. In our study, we included CACDNS participants aged 18 years or older, who had complete data on demographic and lifestyle factors, dietary intake, medical history, physical examination and laboratory results (*n* = 69,909). We further excluded individuals with outlying total energy intakes (<800 kilocalories per day (kcal/day) or >4500 kcal/day) (*n* = 2169), pregnant women (*n* = 141), nursing mothers (*n* = 153) and cancer patients (*n* = 1019). The final sample size of participants included in this study was 66,427.

Written informed consent was obtained by all participants at the beginning of the survey. All procedures involving human subjects were approved by the Ethics Committee of the Chinese Center for Disease Control and Prevention (approval number: 201519-B).

### 2.2. Dietary Assessment

The dietary intake for each participant was assessed using the three-day (two weekdays and one weekend day) food record method, combined with the three-day household condiments weighing method. All the interviewers were public health physicians from the local centers for Disease Control and Prevention or community health centers who had received standard training courses on the recording of dietary information. The interviewers used standard forms for the dietary recalls with picture aids and food models during household interviews. During the survey, participants were asked not to change dietary or lifestyle habits. Nutrient intakes, including intakes of dietary fiber, animal protein and total energy, were calculated according to dietary data and the Chinese Food Composition Table [26]. Because only a few Chinese adults (0.7%) were using nutrient supplements, we excluded dietary supplements and drugs from the dietary intake assessment [27]. Different sources of dietary fiber intake were identified by food codes. Total dietary fiber intake comes from grains, legumes, vegetables, fruits and some other foods (tubers, nuts, sauces, etc.).

### 2.3. Anthropometric and Laboratory Measurements

Physical examinations were performed by trained medical staff following the standardized procedures. Body weight was measured by using calibrated electronic digital scales with a precision of 0.01 kg, and height was measured by using height measuring bars with a precision of 0.1 cm. Body mass index (BMI) was then calculated as kg/m^2^. An automatic measurement device (Omron HBP-1300; OMRON Healthcare, Hoofddorp, The Netherlands) was used to measure systolic and diastolic blood pressure three times with a 1-min interval between each measurement. The average of three measurements was used in the analysis. 

All participants were invited to provide 12 h overnight fasting blood samples. Blood samples were centrifuged and separated into plasma and serum within 0.5–1.0 h after blood collection, and frozen at −80 °C for subsequent testing. SUA, triglycerides, fasting blood glucose, high-density lipoprotein cholesterol, total cholesterol and low-density lipoprotein cholesterol levels were measured using a Hitachi Automatic Analyzer 7600 (Hitachi Co., Tokyo, Japan).

### 2.4. Potential Confounders

Information on age, gender, region, education, marital status, smoking status, drinking status, physical activity level and medical histories was collected from each participant by using an interviewer-administered questionnaire. Educational attainment was divided into three categories: less than high school, high school and more than high school. Marital status was coded as living single (separated, divorced or widowed) and not single (married or living with partner). Physical activity was summed and expressed in metabolic equivalent (MET) minutes per week, and then categorized into three grades: sedentary (MET < 600), moderate (600 ≤ MET ≤ 3000) or vigorous (MET > 3000) [28,29]. Smoking status and drinking status were categorized into yes (current or former) or no (never). HUA was defined as SUA level ≥ 7.0 mg/dL in men and ≥6.0 mg/dL in women [30]. Diabetes was defined as self-reported history of diabetes mellitus or fasting glucose ≥ 7.0 mmol/L. Hypertension was defined as self-reported history of hypertension, or an average SBP or an average SBP ≥ 140 mm Hg or DBP ≥90 mm Hg. Dyslipidemia was defined as total cholesterol (TC) level ≥ 5.70 mmol/L, high-density lipoprotein cholesterol (HDL-C) level < 1.04 mmol/L or current use of antihyperlipidemic drugs. Urologic disease, such as kidney stones, prostatitis, chronic nephritis was assessed by self-reporting.

### 2.5. Statistical Analysis

Baseline characteristics of the study participants were described as median (interquartile range (IQR)) for continuous variables and number (percentage) for categorical variables. Differences between the HUA and non-HUA groups were analyzed by Mann–Whitney U test for continuous variables and chi-square test for categorical variables. Participants were further classified into quartiles according to total dietary fiber intake from the lowest (1st quartile) to the highest (4th quartile). *p* value for trend across the quartiles was then calculated by generalized linear model for continuous variables, and Mantel–Haenszel chi-square test for categorical variables. Multilevel models were included in our analysis to avoid potential aggregation bias, as the occurrence of HUA might aggregate in families due to a similar genetic and lifestyle background. The family aggregation was used as the random-effect variable and the dietary fiber intakes were considered as the independent fixed-effect variables. On the basis of this, mixed-effects linear regression of dietary fiber intake on SUA levels and mixed-effects logistic regression of dietary fiber intake on hyperuricemia were conducted. Total dietary fiber, cereal fiber and vegetable fiber were grouped according to the quartile of intake, and the lowest intake group was used as the control group. As the proportion of participants without dietary fiber intake from legume (45.5%) and fruit (65%) is relatively large, 0% was set as the reference group intake, and the rest was divided into tertiles. *p* values for trend (*p*-trend) were calculated by using the median within each group to evaluate the ordered relation across groups of the dietary exposures for both the continuous (SUA) and binary (hyperuricemia) outcomes. All multivariable regression models were adjusted for these covariates: Model 1 was adjusted for age and gender; Model 2 was additionally adjusted for BMI, region, race, education background, marital status, physical activity level, smoking status, drinking status, total energy intake and animal protein intake; Model 3 was further adjusted for hypertension, diabetes, dyslipidemia and urologic disease. A *p*-value ≤ 0.05 was defined as statistical significance. Statistical analyses were performed using the R software version 3.6.3. 

## 3. Results

**Figure 1:**
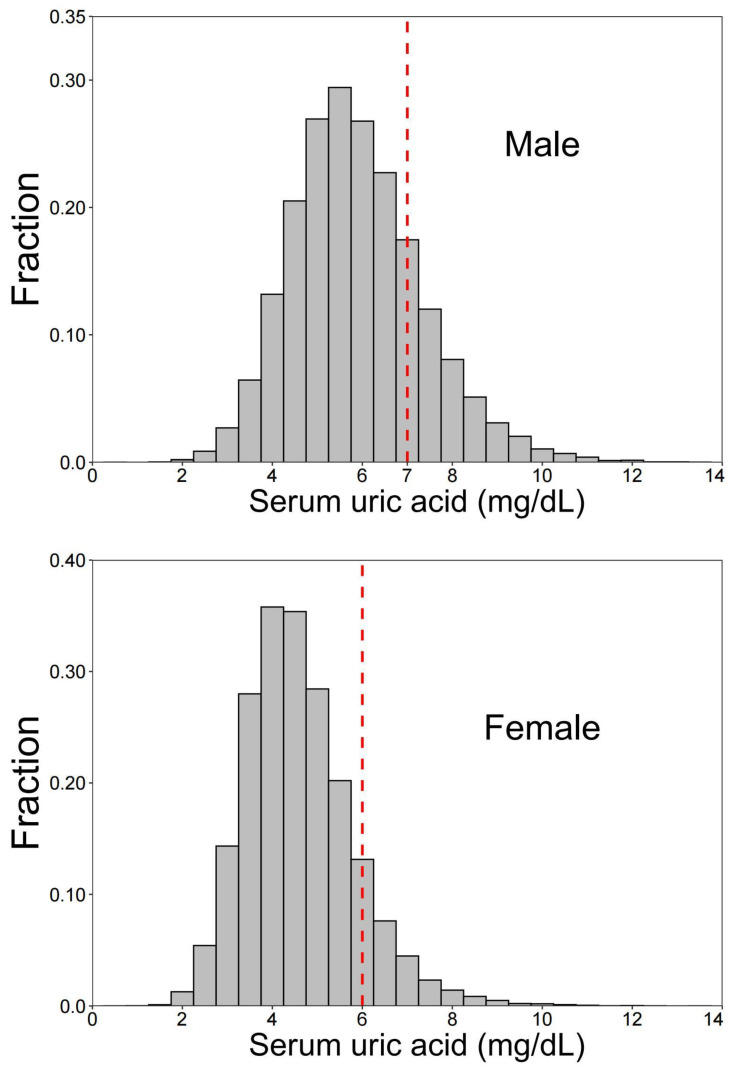
Distribution in serum concentrations of uric acid among 66,427 participants aged 18 years and above, CACDNS 2015. The red-dashed line indicates the cut-off values of uric acid level for hyperuricemia diagnosis, which for male is 7.0 mg/dL in men and 6.0 mg/dL in women.

**Figure 2:**
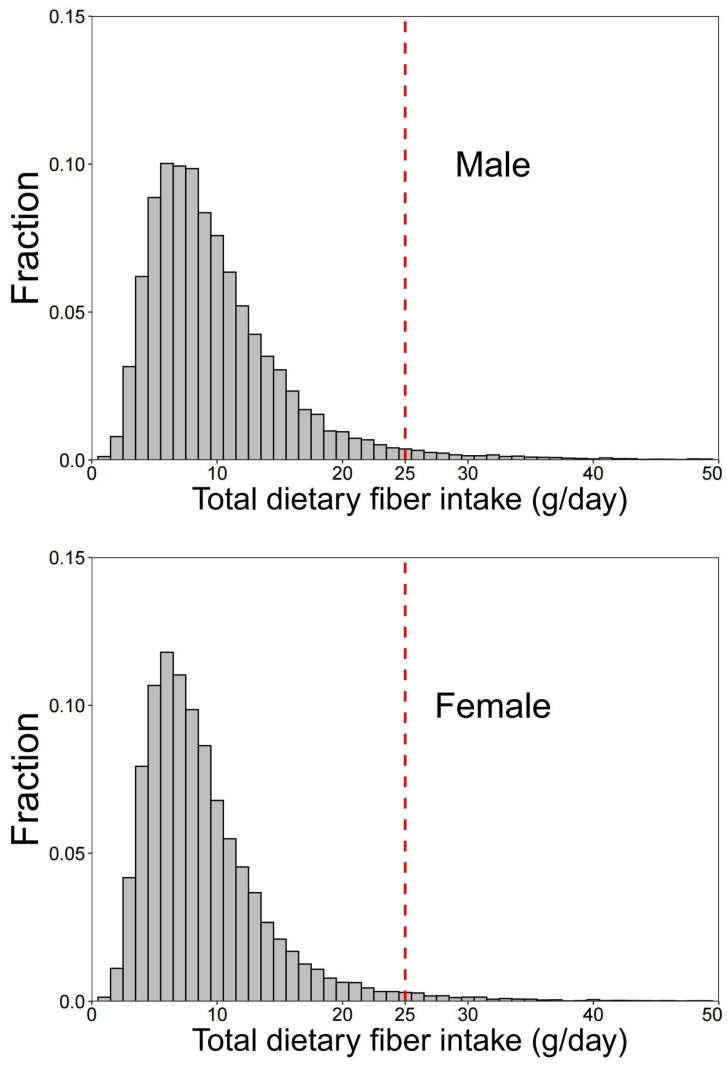
Distribution in intake of total dietary fiber among 66,427 participants aged 18 years and above, CACDNS 2015. The red-dashed line indicates the recommended daily fiber intake of 25 g/day.

A total of 66,427 adult subjects were enrolled in this study, including 31,920 males and 34,507 females. The overall prevalence of HUA in the entire sample was 14% (18% in men; 10.3% in women) (Figure 1). The median (IQR) of total dietary fiber intake was 8.19 (5.8–11.59) g/day. Only 2.5% of the participants (2.8% in men; 2.1% in women) reached the minimum recommended daily fiber intake of 25 g/day [31] (Figure 2).

Table 1 shows the result of comparing the indicators between HUA and non-hyperuricemia for both sexes. Compared with the controls, both men and women with HUA were more likely to be urban residents, live alone, consume alcohol, have hypertension, diabetes, dyslipidemia and urologic disease as well as have higher levels of BMI and dietary intake of animal protein, vegetable fiber and fruit fiber. Physical activity, dietary intakes of total and cereal fiber were significantly higher in subjects without hyperuricemia. The male participants with HUA were more likely to be younger, non-Han Chinese, and be educated past high school. Female participants with HUA were more likely to be older, less than high school educated, smokers and have lower total energy intake.

The characteristics of the study participants across quartiles of total dietary fiber intake are presented in Table 2. Participants with higher dietary fiber intake were more likely to be men, aged 45–59 years, rural residents, Han Chinese, high school educated, married or living with their partners, less physically active, non-smokers, non-drinkers and without diabetes, dyslipidemia and urologic disease. With increased dietary fiber, BMI, total energy, animal protein, grain fiber, legume fiber, vegetable fiber and fruit fiber intakes, the level of uric acid decreased.

Table 3 shows the results for the multiple linear regression, which assessed the association between the dietary fiber intake and change in SUA levels. After adjustment for multiple potential confounders (Model 3), a significantly inverse association was observed between total fiber intake with SUA levels. Compared with the first quartile, the β and 95% CI of Q2, Q3, and Q4 were −0.03 (−0.05, −0.02), −0.06 (−0.08, −0.05), and −0.06 (−0.08, −0.04) (*p*-trend < 0.001). The dietary fiber was further categorized according to the food source. A negative linear association was found between cereal fiber intake and SUA levels. Compared with the first quartile, the β and 95% CI of Q2, Q3 and Q4 were −0.04 (−0.05, −0.03), −0.11 (−0.13, −0.1) and −0.18 (−0.2, −0.16) in Model 3 (*p*-trend < 0.001). No significant linear association between legume fiber, vegetable fiber and fruit fiber with SUA levels was observed in the fully adjusted model.

Multivariable-adjusted ORs and 95% CI for HUA according to dietary intake levels are depicted in Table 4. After adjusting for all potential confounders (Model 3), more intake of total dietary fiber presented beneficial effects. Individuals in the highest quartile of total dietary fiber intake were 13% less likely to have HUA than those in the lowest quartile. Compared with the lowest quartile of total dietary fiber intake, the ORs and 95% CI for HUA were 0.93 (0.9, 0.96), 0.85 (0.82, 0.88) and 0.88 (0.84, 0.91), and *p* for trend was 0.001. The different sources of dietary fiber were further categorized, and cereal dietary fiber was found to be strongly and negatively associated with HUA. Compared with the lowest quartile of cereal fiber intake, the ORs and 95% CI for HUA were 0.94 (0.91, 0.97), 0.78 (0.75, 0.82) and 0.67 (0.63, 0.71), and *p* for trend was less than 0.001. No significant association was observed between intake of legume fiber, vegetable fiber and fruit fiber with the risk of HUA after full adjustment.

## 4. Discussion

In this nationally representative cross-sectional study, we observed that total dietary fiber and cereal fiber intake was negatively associated with SUA levels and HUA among Chinese adults. These associations remained after adjusting for potential confounders, including age, gender, BMI, region, race, education background, marital status, physical activity level, smoking status, drinking status, total energy intake, animal protein intake, hypertension, diabetes, dyslipidemia and urologic disease. 

To the best of our knowledge, this is the largest population-based study on the association of dietary fiber intake with SUA levels and HUA, and the first study on this topic in Chinese adults. The beneficial effects of dietary fiber intake on the hyperuricemia have previously been reported by several epidemiological studies. The data from NHANES 1999–2004 indicated that increased dietary fiber intake was significantly associated with decreased HUA risk in adults aged 20 to 80 years, without diabetes, cancer or heart disease [22]. Another study analyzed NHANES data from 2005–2008 on adolescents aged 13 to 18 years and revealed that a higher intake of fiber correlated with lower SUA levels [20]. Results from the Nutrition and Health Survey in Taiwan 1993–1996 showed that dietary fiber intake was significantly and negatively associated with HUA in adults [21]. A cross-sectional study on Koreans compared the nutrient intake and diet quality between HUA subjects and controls, and found that the HUA subjects had a lower intake of dietary fiber than controls [32]. Moreover, a randomized, double-blind, placebo-controlled clinical study from the United States reported that a soluble, highly viscous functional fiber supplement, composed of three water-soluble polysaccharides, could significantly reduce SUA in the healthy subjects [33]. However, in a cross-sectional analysis, Caucasian adults with diets from different food traditions showed different results: dietary fiber intake was significantly and negatively associated with SUA concentrations in the Australian but not in the Norwegian populations [24]. The inconsistent results may be due to the daily fiber intake in Norway participants being much lower than the corresponding value in Australia, while our results showed that the association between dietary fiber with SUA may be very weak at low intakes. 

We further explored the association of dietary fiber from different food sources with SUA levels and HUA, and found a higher cereal fiber intake was associated with lower SUA levels and risk of HUA in Chinese adults. This result is largely in accordance with the results from NHANES 2009–2014, which also observed an inverse association between cereal fiber intake and risk of HUA in the U.S. adults [23]. Cereals accounted for nearly 40% of total dietary fiber and comprised the major source of dietary fiber for Chinese adults [34]. However, the consumption of grains and dietary fiber in the Chinese population has been continuously declining since 1982 and the consumption of refined grains has increased [34]. Cereals, breads and grain mixtures with higher contents of both dietary fiber and whole grain should be recommended by nutrition educators and policy makers to consumers across all eating occasions in order to increase cereal fiber intakes among the Chinese population.

In this study, we did not observe a significant association between SUA levels and risk of HUA with consumption of dietary fiber from legumes, fruits and vegetables. There are several possible explanations. Animal experiments have suggested that the beneficial effect on SUA is stronger for dietary fiber with viscosity than for water-insoluble dietary fiber based on experimental overproduction-type HUA in rats [19]. Thus, the differences in fiber composition in cereals, fruits and vegetables are likely to be one of the causes of the different abilities to reduce uric acid levels [35]. Moreover, legumes and some vegetables are rich in purines and most of the fruits contain fructose, therefore the influence of purine or fructose may counteract the protective effects that dietary fiber from legumes, vegetables and fruit provide for HUA, to some extent. Overall, the exact reasons for these observations remain unclear and require further investigation.

Although the exact mechanism for the benefit effects of dietary fiber intake on HUA remains largely unknown, it may be a result of changes in nutrient absorption, intestinal viscosity, rate of passage, and production of short chain fatty acids and gut hormones [36]. The viscosity and bulkiness of dietary fiber may interfere with the absorption of purine or adenine in the digestive system, thereby reducing the production of uric acid [19]. Dietary fiber has been shown to be positively associated with kidney function and increased gut transit rate and fecal bulk, thus leading to increased uric acid excretion [17,18]. Higher insulin levels and insulin resistance can contribute to increased uric acid synthesis and reduced renal excretion of urate [37,38,39]. Dietary fibers can accelerate secretion of glucose-dependent insulinotropic polypeptide (GIP) and be fermented by gut microbiota to produce short chain fatty acids (SCFAs), which could attenuate insulin responses and improve insulin sensitivity [36], and eventually reduce urate levels. In addition, dietary fiber intake adversely affects markers for inflammation [38], which has been positively linked to HUA [39]. It is possible that inflammation is a potential meditator between dietary fiber intake and SUA levels. The detailed mechanism regarding how dietary fiber, especially cereal fiber, affects HUA, warrants future studies.

Our study has several strengths. First, this is the first study to assess the association of dietary fiber intake from different food sources with SUA levels and the risk of HUA among Chinese adults, and to use a large (66,427 participants) and nationally representative sample. The results are therefore robust. Second, the negative association of total fiber intake and cereal fiber intake with SUA levels and HUA persisted and was significant after adjustment for multiple potential confounding variables. Third, the use of standardized protocols and data collection procedures in this study, along with training and strict quality control for all participants, ensured the reliability of results for this study.

There are also some limitations to this study. We can not confirm the causal relationship of dietary fiber intake with SUA levels and HUA due to the cross-sectional nature of this study. Prospective and longitudinal studies would be important to confirm those conclusions. In addition, since we used the 24 h dietary recall method to estimate dietary intake levels, long-term fiber intake may not be accurately described. However, compared with the food frequency questionnaire, the 24 h recall provided more detailed information about the type and amount of food eaten, thus may reduce the risk of underestimating or overestimating micro-nutrient intake levels.

## 5. Conclusions

In summary, we observed an inverse association of total and cereal fiber intake with SUA levels and the risk of HUA among the Chinese adult population. Effective public education programs should be developed and implemented to increase the intake of dietary fiber, especially cereal dietary fiber among Chinese adults.

## Figures and Tables

**Table 1:** Sample characteristics of participants with or without hyperuricemia (*n* = 66,427).

Characteristics	Male		*p*	Female		*p*
	Non-Hyperuricemia	Hyperuricemia		Non-Hyperuricemia	Hyperuricemia	
	(*n* = 26,159)	(*n* = 5761)		(*n* = 30,949)	(*n* = 3558)	
Age (years)	55 (45, 65)	53 (42, 64)	<0.001	52 (43, 62)	59 (49,67)	<0.001
Age group (years)			<0.001			<0.001
<45	6216 (23.8%)	1689 (29.3%)		8721 (28.2%)	635 (17.8%)	
45–59	9519 (36.4%)	1966 (34.1%)		12,027 (38.9%)	1187 (33.4%)	
≥60	10,424 (39.8%)	2106 (36.6%)		10,201 (33%)	1736 (48.8%)	
Region			<0.001			<0.001
City	10,106 (38.6%)	2577 (44.7%)		12,609 (40.7%)	1713 (48.1%)	
Rural	16,053 (61.4%)	3184 (55.3%)		18,340 (59.3%)	1845 (51.9%)	
Ethic group			0.014			0.489
Han	23,344 (89.2%)	5077 (88.1%)		27,545 (89%)	3153 (88.6%)	
Others	2815 (10.8%)	684 (11.9%)		3404 (11%)	405 (11.4%)	
Education			<0.001			<0.001
<High School	10,833 (41.4%)	2111 (36.6%)		17,256 (55.8%)	2157 (60.6%)	
High School	13,419 (51.3%)	3067 (53.2%)		11,534 (37.3%)	1201 (33.8%)	
>High School	1907 (7.3%)	583 (10.1%)		2159 (7%)	200 (5.6%)	
Marital status			<0.001			<0.001
Single	1963 (7.5%)	534 (9.3%)		2376 (7.7%)	395 (11.1%)	
Not single	24,196(92.5%)	5227 (90.7%)		28,573 (92.3%)	3163 (88.9%)	
Physical Activity Level			<0.001			<0.001
Sedentary	4856 (18.6%)	1163 (20.2%)		4617 (14.9%)	584 (16.4%)	
Moderate	7512 (28.7%)	1891 (32.8%)		10,263 (33.2%)	1184 (33.3%)	
Vigorous	13,791 (52.7%)	2707 (47%)		16,069 (51.9%)	1790 (50.3%)	
Smoking (yes)	17,367 (66.4%)	3836 (66.6%)	0.776	1065 (3.4%)	168 (4.7%)	<0.001
Drinking (yes)	14,846 (56.8%)	3680 (63.9%)	<0.001	5149 (16.6%)	649 (18.2%)	0.015
Hypertension (yes)	11,156 (42.6%)	3080 (53.5%)	<0.001	11,940 (38.6%)	2039 (57.3%)	<0.001
Diabetes (yes)	2345 (9%)	532 (9.2%)	0.517	2420 (7.8%)	537 (15.1%)	<0.001
Dyslipidemia (yes)	6385 (24.4%)	2604 (45.2%)	<0.001	7196 (23.3%)	1567 (44%)	<0.001
Urologic disease (yes)	3280 (12.5%)	814 (14.1%)	0.001	2373 (7.7%)	395 (11.1%)	<0.001
BMI (kg/m^2^)	23.7 (21.4, 26.1)	25.2 (22.8, 27.7)	<0.001	23.8 (21.5, 26.3)	25.6 (23.2, 28.3)	<0.001
SUA (mg/dL)	5.4 (4.7, 6.2)	7.9 (7.5, 8.6)	<0.001	4.3 (3.7, 5)	6.8 (6.4, 7.3)	<0.001
Intakes of						
Total energy (kcal/day)	1874 (1502.2, 2305)	1860.8 (1490.2, 2307.2)	0.276	1547 (1252.8, 1890.5)	1528.3 (1232.1, 1877.6)	0.036
Animal protein (g/day)	16.3 (7.3, 28.8)	22.2 (11.9, 35.8)	<0.001	13.7 (6.3, 24.2)	17.8 (9.2, 28.8)	<0.001
Total fiber (g/day)	8.8 (6.2, 12.4)	8 (5.7, 11.5)	<0.001	7.9 (5.6, 11)	7.4 (5.2, 10.7)	<0.001
Cereal fiber (g/day)	2.8 (1.8, 5)	2.2 (1.5, 3.5)	<0.001	2.3 (1.5, 4)	2 (1.3, 3.1)	<0.001
Legume fiber (g/day)	0.1 (0, 0.4)	0.1 (0, 0.5)	0.188	0.1 (0, 0.4)	0.1 (0, 0.4)	0.579
Vegetable fiber (g/day)	2.6 (1.5, 4.1)	2.8 (1.7, 4.5)	<0.001	2.4 (1.4, 3.9)	2.7 (1.6, 4.3)	<0.001
Fruit fiber (g/day)	0 (0, 0.3)	0 (0, 0.4)	<0.001	0 (0, 0.6)	0 (0, 0.7)	0.01

BMI, body mass index; SUA, serum uric acid. Continuous variables are described as median (IQR); categorical variables are described as participants (percentage). *p* values were calculated by Mann–Whitney U test for continuous variables, and chi-square test for categorical variables.

**Table 2:** Sample characteristics of participants according to quartile of dietary fiber intake (*n* = 66,427).

Characteristics	Dietary Fiber Intake (mg/day)				*p*-Trend
	Q1 (≤5.85)	Q2 (5.86–8.25)	Q3 (8.26–11.65)	Q4 (≥11.66)	
n	16,915	16,742	16,385	16,385	
male	7233 (42.8%)	7661 (45.8%)	8103 (49.5%)	8923 (54.5%)	<0.001
Age (years)	54 (44, 65)	54 (44, 64)	53 (44, 63)	53 (44, 63)	<0.001
Age group					<0.001
<45	4450 (26.3%)	4346 (26%)	4342 (26.5%)	4123 (25.2%)	
45–59	5867 (34.7%)	6161 (36.8%)	6217 (37.9%)	6454 (39.4%)	
≥60	6598 (39%)	6235 (37.2%)	5826 (35.6%)	5808 (35.4%)	
Region					<0.001
City	6120 (36.2%)	6888 (41.1%)	7099 (43.3%)	6898 (42.1%)	
Rural	10,795 (63.8%)	9854 (58.9%)	9286 (56.7%)	9487 (57.9%)	
Ethic group					<0.001
Han	14,321 (84.7%)	15,026 (89.8%)	14,913 (91%)	14,859 (90.7%)	
Others	2594 (15.3%)	1716 (10.2%)	1472 (9%)	1526 (9.3%)	
Education					<0.001
<High School	9486 (56.1%)	8396 (50.1%)	7429 (45.3%)	7046 (43%)	
High School	6465 (38.2%)	7170 (42.8%)	7622 (46.5%)	7964 (48.6%)	
>High School	964 (5.7%)	1176 (7%)	1334 (8.1%)	1375 (8.4%)	
Marital status					<0.001
Single	1620 (9.6%)	1399 (8.4%)	1201 (7.3%)	1048 (6.4%)	
Not single	15,295 (90.4%)	15,343 (91.6%)	15,184 (92.7%)	15,337 (93.6%)	
Physical Activity Level					<0.001
Sedentary	3118 (18.4%)	2897 (17.3%)	2742 (16.7%)	2463 (15%)	
Moderate	5328 (31.5%)	5350 (32%)	5131 (31.3%)	5041 (30.8%)	
Vigorous	8469 (50.1%)	8495 (50.7%)	8512 (51.9%)	8881 (54.2%)	
Smoking (yes)	5290 (31.3%)	5429 (32.4%)	5646 (34.5%)	6071 (37.1%)	<0.001
Drinking (yes)	5550 (32.8%)	5812 (34.7%)	6246 (38.1%)	6716 (41%)	<0.001
Hypertension (yes)	7088 (41.9%)	7234 (43.2%)	6883 (42%)	7010 (42.8%)	0.396
Diabetes (yes)	1414 (8.4%)	1464 (8.7%)	1471 (9%)	1485 (9.1%)	0.017
Dyslipidemia (yes)	4396 (26%)	4521 (27%)	4325 (26.4%)	4510 (27.5%)	0.009
Urologic disease (yes)	1641 (9.7%)	1739 (10.4%)	1684 (10.3%)	1798 (11%)	<0.001
Hyperuricemia (yes)	14,202 (84%)	14,328 (85.6%)	14,311 (87.3%)	14,267 (87.1%)	<0.001
BMI (kg/m^2^)	23.5 (21.2, 26)	23.9 (21.6, 26.4)	24.1 (21.8, 26.6)	24.3 (22, 26.8)	<0.001
SUA (mg/dL)	5.1 (4.2, 6.2)	5 (4.1, 6.1)	5 (4.1, 6)	5 (4.2, 6.1)	<0.001
Intakes of					
Total energy (kcal/day)	1359.5 (1106.9, 1692)	1575.1 (1302.8, 1919.9)	1772 (1486.2, 2124.5)	2085.9 (1735.1, 2517.8)	<0.001
Animal protein (g/day)	15.1 (7.3, 25.8)	15.1 (6.8, 26.7)	15.7 (7, 27.7)	16.5 (7.3, 29)	<0.001
Cereal fiber (g/day)	1.6 (1.2, 2.3)	2.4 (1.6, 3.7)	3.2 (1.9, 5.3)	3.9 (2.1, 7.3)	<0.001
Legume fiber (g/day)	0 (0, 0.2)	0.1 (0, 0.3)	0.1 (0, 0.6)	0.2 (0, 1.7)	<0.001
Vegetable fiber (g/day)	1.6 (1, 2.4)	2.5 (1.5, 3.6)	3 (1.8, 4.7)	4.1 (2.3, 7.4)	<0.001
Fruit fiber (g/day)	0 (0, 0)	0 (0, 0.3)	0 (0, 0.7)	0 (0, 1.2)	<0.001

BMI, body mass index; SUA, serum uric acid. Continuous variables are described as median (IQR); categorical variables are described as participants (percentage). *p*-trends were calculated by generalized linear model for continuous variables, and Mantel–Haenszel χ^2^ test for categorical variables.

**Table 3:** Multivariable linear associations between dietary fiber intake and serum uric acid (*n* = 66,427).

Dietary Fiber Intake		Model 1	Model 2	Model 3
		β (95% CI)	β (95% CI)	β (95% CI)
Total fiber (g/day)				
	≤5.85	1.00 (ref.)	1.00 (ref.)	1.00 (ref.)
	5.86–8.25	−0.03 (−0.04, −0.01)	−0.03 (−0.04, −0.02)	−0.03 (−0.05, −0.02)
	8.26–11.65	−0.06 (−0.07, −0.04)	−0.07 (−0.08, −0.05)	−0.06 (−0.08, −0.05)
	≥11.66	−0.06 (−0.07, −0.04)	−0.06 (−0.08, −0.04)	−0.06 (−0.08, −0.04)
	*p*-trend	<0.001	<0.001	<0.001
Cereal fiber (g/day)				
	≤2.36	1.00 (ref.)	1.00 (ref.)	1.00 (ref.)
	2.37–3.75	−0.03 (−0.04, −0.02)	−0.03 (−0.05, −0.02)	−0.04 (−0.05, −0.03)
	3.76–6	−0.1 (−0.12, −0.09)	−0.11 (−0.13, −0.09)	−0.11 (−0.13, −0.1)
	≥6.1	−0.18 (−0.2, −0.16)	−0.18 (−0.2, −0.15)	−0.18 (−0.2, −0.16)
	*p*-trend	<0.001	<0.001	<0.001
Legume fiber (g/day)				
	0	1.00 (ref.)	1.00 (ref.)	1.00 (ref.)
	0.01–0.2	0.01 (0, 0.03)	0 (−0.01, 0.02)	0.01 (0, 0.02)
	0.21–1.13	−0.01 (−0.02, 0)	−0.02 (−0.03, −0.01)	−0.01 (−0.03, 0)
	≥1.14	0.02 (0, 0.03)	0.02 (0.01, 0.04)	0.03 (0.01, 0.04)
	*p*-trend	0.345	0.075	0.051
Vegetable fiber (g/day)				
	≤1.68	1.00 (ref.)	1.00 (ref.)	1.00 (ref.)
	1.69–3.1	0.01 (−0.01, 0.02)	0 (−0.01, 0.01)	0 (−0.01, 0.01)
	3.11–5.02	0.01 (−0.01, 0.02)	0 (−0.02, 0.01)	0 (−0.02, 0.01)
	≥5.03	0.02 (0.01, 0.04)	0 (−0.02, 0.02)	0 (−0.01, 0.02)
	*p*-trend	0.205	0.904	0.869
Fruit fiber (g/day)				
	0	1.00 (ref.)	1.00 (ref.)	1.00 (ref.)
	0.01–0.58	−0.02 (−0.05, 0)	−0.02 (−0.04, 0.01)	−0.02 (−0.04, 0.01)
	0.59–1.56	0 (−0.02, 0.03)	0.01 (−0.01, 0.04)	0.01 (−0.01, 0.04)
	≥1.57	0.01 (−0.01, 0.04)	0.01 (−0.02, 0.03)	0.01 (−0.01, 0.04)
	*p*-trend	0.043	0.266	0.296

Model 1 was adjusted for age and gender. Model 2 was additionally adjusted for BMI, region, race, education background, marital status, physical activity level, smoking status, drinking status, total energy intake and animal protein intake. Model 3 was further adjusted for hypertension status, diabetes status, dyslipidemia status and urologic disease status.

**Table 4:** Multivariate-adjusted ORs (95% CIs) of hyperuricemia according to dietary fiber intake (*n* = 66,427).

Dietary Fiber Intake		Model 1	Model 2	Model 3
		OR (95% CI)	OR (95% CI)	OR (95% CI)
Total fiber (g/day)				
	≤5.85	1.00 (ref.)	1.00 (ref.)	1.00 (ref.)
	5.86–8.25	0.95 (0.92, 0.98)	0.94 (0.91, 0.97)	0.93 (0.9, 0.96)
	8.26–11.65	0.86 (0.83, 0.89)	0.85 (0.81, 0.88)	0.85 (0.82, 0.88)
	≥11.66	0.89 (0.86, 0.92)	0.88 (0.85, 0.92)	0.88 (0.84, 0.91)
	*p*-trend	<0.001	<0.001	0.001
Cereal fiber (g/day)				
	≤2.36	1.00 (ref.)	1.00 (ref.)	1.00 (ref.)
	2.37–3.75	0.96 (0.93, 0.99)	0.95 (0.92, 0.98)	0.94 (0.91, 0.97)
	3.76–6	0.81 (0.78, 0.84)	0.79 (0.76, 0.82)	0.78 (0.75, 0.82)
	≥6.1	0.68 (0.65, 0.72)	0.68 (0.64, 0.72)	0.67 (0.63, 0.71)
	*p*-trend	<0.001	<0.001	<0.001
Legume fiber (g/day)				
	0	1.00 (ref.)	1.00 (ref.)	1.00 (ref.)
	0.01–0.2	1.02 (0.99, 1.06)	1.01 (0.98, 1.04)	1.02 (0.98, 1.05)
	0.21–1.13	0.99 (0.96, 1.02)	0.98 (0.95, 1.02)	0.99 (0.96, 1.03)
	≥1.14	1.02 (0.98, 1.05)	1.04 (1, 1.08)	1.05 (1, 1.09)
	*p*-trend	0.736	0.244	0.248
Vegetable fiber (g/day)				
	≤1.68	1.00 (ref.)	1.00 (ref.)	1.00 (ref.)
	1.69–3.1	1.02 (0.98, 1.05)	1 (0.97, 1.04)	1.01 (0.97, 1.04)
	3.11–5.02	1 (0.96, 1.03)	0.99 (0.95, 1.02)	0.98 (0.95, 1.02)
	≥5.03	1.03 (0.99, 1.07)	0.99 (0.95, 1.03)	1.01 (0.97, 1.05)
	*p*-trend	0.559	0.776	0.982
Fruit fiber (g/day)				
	0	1.00 (ref.)	1.00 (ref.)	1.00 (ref.)
	0.01–0.58	1.03 (0.98, 1.1)	1.04 (0.98, 1.11)	1.04 (0.98, 1.11)
	0.59–1.56	1.04 (0.98, 1.1)	1.06 (1, 1.13)	1.06 (1, 1.13)
	≥1.57	1.06 (1, 1.12)	1.05 (0.99, 1.11)	1.06 (1, 1.12)
	*p*-trend	0.486	0.214	0.264

Model 1 was adjusted for age and gender. Model 2 was additionally adjusted for BMI, region, race, education background, marital status, physical activity level, smoking status, drinking status, total energy intake and animal protein intake. Model 3 was further adjusted for hypertension status, diabetes status, dyslipidemia status and urologic disease status.

## Data Availability

The data are not allowed to be disclosed according to the National Institute for Nutrition and Health, Chinese Center for Disease Control and Prevention.

## Abbreviations

SUA	serum uric acid
HUA	hyperuricemia
BMI	body mass index
